# A new method assessing predicted and achieved mandibular tooth movement in adults treated with clear aligners using CBCT and individual crown superimposition

**DOI:** 10.1038/s41598-023-31339-8

**Published:** 2023-03-11

**Authors:** Abdulraheem A. Alwafi, Alan G. Hannam, Edwin H. Yen, Bingshuang Zou

**Affiliations:** 1 Department of Oral Health Science, Faculty of Dentistry, University of British Columbia, 2199 Wesbrook Mall, Vancouver, BC V6T 1Z3 Canada; 2 Department of Dental Public Health, Faculty of Dentistry, King Abdulaziz University, Jeddah, Saudi Arabia

**Keywords:** Anatomy, Medical research

## Abstract

The purpose of this study was to demonstrate a new method for quantifying the difference between predicted and achieved tooth movement with Invisalign using stable three-dimensional (3D) mandibular landmarks and dental superimposition. Cone-beam computed tomography (CBCT) scans before (T1) and after (T2) the first series of aligners, their corresponding digital models (ClinCheck initial of the first series as T1 and ClinCheck initial of the refinement series as T2), and the ClinCheck final model of the first series as the predicted were obtained from 5 patients treated with non-extraction Invisalign therapy. After segmentation of the mandible and its dentition, T1 and T2 CBCTs were superimposed on stable anatomic structures (Pogonion and bilateral mental foramen) along with the pre-registered ClinCheck models. The 3D prediction differences between the predicted and achieved tooth position for 70 teeth with four types (incisor, canine, premolar and molar) were measured using a combination of software. The method employed in this study was tested to be reliable and repeatable with a very high intraclass correlation coefficient (ICC) for both intra- and inter-examiner reliability. Premolar Phi (rotation), Incisor Psi (mesiodistal angulation), and Molar Y (mesiodistal translation) showed a significant prediction difference (P < 0.05), which is also clinically relevant. The method involving CBCT and individual crown superimposition to measure the 3D positional changes in the mandibular dentition is a robust and novel one. While, our finding in terms of the predictability of Invisalign treatment in the mandibular dentition mainly served as a crude, cursory examination, which warrants further and more rigorous investigations. With this novel methodology, it is possible to measure any amount of 3D tooth position difference in the mandibular dentition either between the simulated and the actual or with treatment and/or growth. Deliberate use of overcorrection of which specific type of tooth movement with clear aligner treatment and to what extent, might be possible with future studies.

## Introduction

Measuring tooth positional changes, either as a result of orthodontic treatment and/or growth or created by a computer software simulating the proposed orthodontic treatment, is challenging and complicated by the difficulty to identify constant reference points. Without these relatively stable landmarks, it is impossible to differentiate between growth, relative movement and treatment effects or predict the accuracy of the orthodontic appliance system. Palatal structures may only be used as reference points in the maxillary dentition. In the maxilla, most studies used the third palatal ruga as the reliable registration landmark for the superimposition of maxillary digital casts^1,2^. Björk was the first to use metallic implants as an alternative comparison point for regional mandibular superimposition^3–5^. In cephalograms, he also described natural stable structures of the maxilla and mandible. The tip of the chin and the following three internal structures in the mandible are deemed stable: (1) the inner cortical structure of the inferior border of the symphysis, (2) detailed structures from the mandibular canal, and (3) the lower contour of the molar germ from the time that mineralization of the crown is visible until the roots begin to form^4^. However, these landmarks are projections of three-dimensional (3D) structures into two-dimensional (2D) lateral films, making them unreliable, except for those located in the midsagittal plane. Using cone-beam computed tomography (CBCT) images, a study group discovered that the Björk registration was not reliable in most 3D mandibular superimposition cases^6^. The displacement of the mandibular canal and other “stable structures” as a result of development were some of the reasons for their argument.

An et al.^7^ have tried to use bilateral mandibular tori, a primarily nodular mass of dense cortical bone in adults as potential stable landmarks for mandibular dental model registration and superimposition. Albeit, mandibular tori are relatively stable within 2 or 3 years of orthodontic treatment in adult patients, the prevalence varies by ethnic origin from 0.2 to 61% ^8^. Furthermore, the simulation software routinely removes the non-dental structures from the digital image, and treatment management portals for clear aligners or fixed appliance systems make it impossible to use these anatomic landmarks for accurate registration. Therefore, mandibular tori can only be utilized in very few studies. Other mandibular structures, such as lingual surfaces of the alveolar process of the anterior and/or posterior teeth^7^ or unmoved teeth^9^, have also served as registration references, but apparently, neither seems to be appropriate as a reference area.

CBCT can provide a reliable 3D image of the cranial skeletal structures using much less radiation than conventional computed tomography (CT) scans^10^. Researchers have attempted to use CBCT to develop a method for 3D regional mandibular superimposition. Park et al. suggested a surface-to-surface matching method based on the basal bone structure of the mandible to evaluate 3D changes in the lower arch^11^. Dai et al. used a similar method in their study^12^. Ruellas et al. stated that using the mandibular body mask (including the mandible without teeth, alveolar bone, rami and condyles) was a reliable reference for 3D regional registration^6^. Nguyen et al. established 3D stable mandibular structures in growing patients with the aid of bone plates and discovered several anterior stable areas (the chin and symphysis regions)^13^. However, it has been debated whether any stable structures posterior to the symphysis area can be found to improve mandibular superimpositions. A recent study by Chen et al. attempted to identify the most stable mandibular landmarks in growing patients using CBCT scans. They found that during a growth period that averaged 4.6 years, ranging from 11.2 to 19.8 years old, the structures that appeared relatively stable and could be used in mandibular regional superimpositions included Pogonion (Pog), landmarks on the inferior part of the internal symphysis, and the mental foramen^14^.

The ability to measure tooth movement or predict treatment outcomes helps treating clinicians visualize treatment outcomes, make real time adjustments to simulations, easily project corrections, have a better judgment on choosing an appropriate treatment plan, build necessary compensations into the virtual treatment plan, provide better interaction and communication with patients, and help with extraction or interproximal reduction decisions. While, previous studies that used 3D superimposition to assess the difference between predicted and achieved mandibular tooth movement are either lacking of stable landmarks^15,16^ or using a common-structured cranial facial coordinate system to express the 3D tooth movement^12^. Therefore, the purpose of this study is to introduce a novel application methodology to assess differences between the predicted and achieved mandibular tooth movement with clear aligner therapy using stable mandibular landmarks and a tooth-specific coordinate system.

## Materials and methods

The informed consent was waived due to the retrospective nature of this study and the research protocol was approved by the Institutional Review Board at the University of British Columbia, Vancouver, Canada (No. H21-02474).

### Participants

All patients receiving orthodontic clear aligner treatment exclusively using Invisalign (Align Technology, Santa Clara, CA, USA) during the period between Jan 2016 and Dec 2018 from a private clinic were selected.

#### Inclusion and exclusion criteria

The main inclusion criteria for patient recruitment were (1) had a permanent dentition with or without third molars before treatment; (2) have completed the first series of clear aligner treatment; (3) had good-quality pre-treatment and post-treatment CBCT scans after the initial series; (4) underwent non-extraction treatment; and (5) had good compliance with the clear aligner treatment according to the chart. The main exclusion criteria were as follows: (1) an initial series of aligners not completed or had a mid-course correction; (2) incomplete or missing radiographic and dental records; (3) the chin was cut out on the CBCT scans; (4) missing tooth except third molars; (5) had an auxiliary appliance before or during aligner treatment other than attachments or elastics; (6) extraction cases; and (7) non-compliant cases.

Finally, 5 patients (including 70 mandibular teeth) were selected and we collected the following records for each patient: (1) Pre-treatment (T1) CBCT; (2) post-treatment (T2, after the initial series, before refinement) CBCT; (3) ClinCheck initial and final stereolithographic (STL) files from the first series served as T1 and ClinCheck predicted digital models respectively; (4) ClinCheck initial STL file from the refinement series served as the T2 digital model.

### Superimposition of the actual and predicted models

To superimpose the T2 achieved model on the ClinCheck predicted model, by using mandibular anatomic reference landmarks on CBCT, the following steps were followed.

#### CBCT segmentation

**Figure 1:**
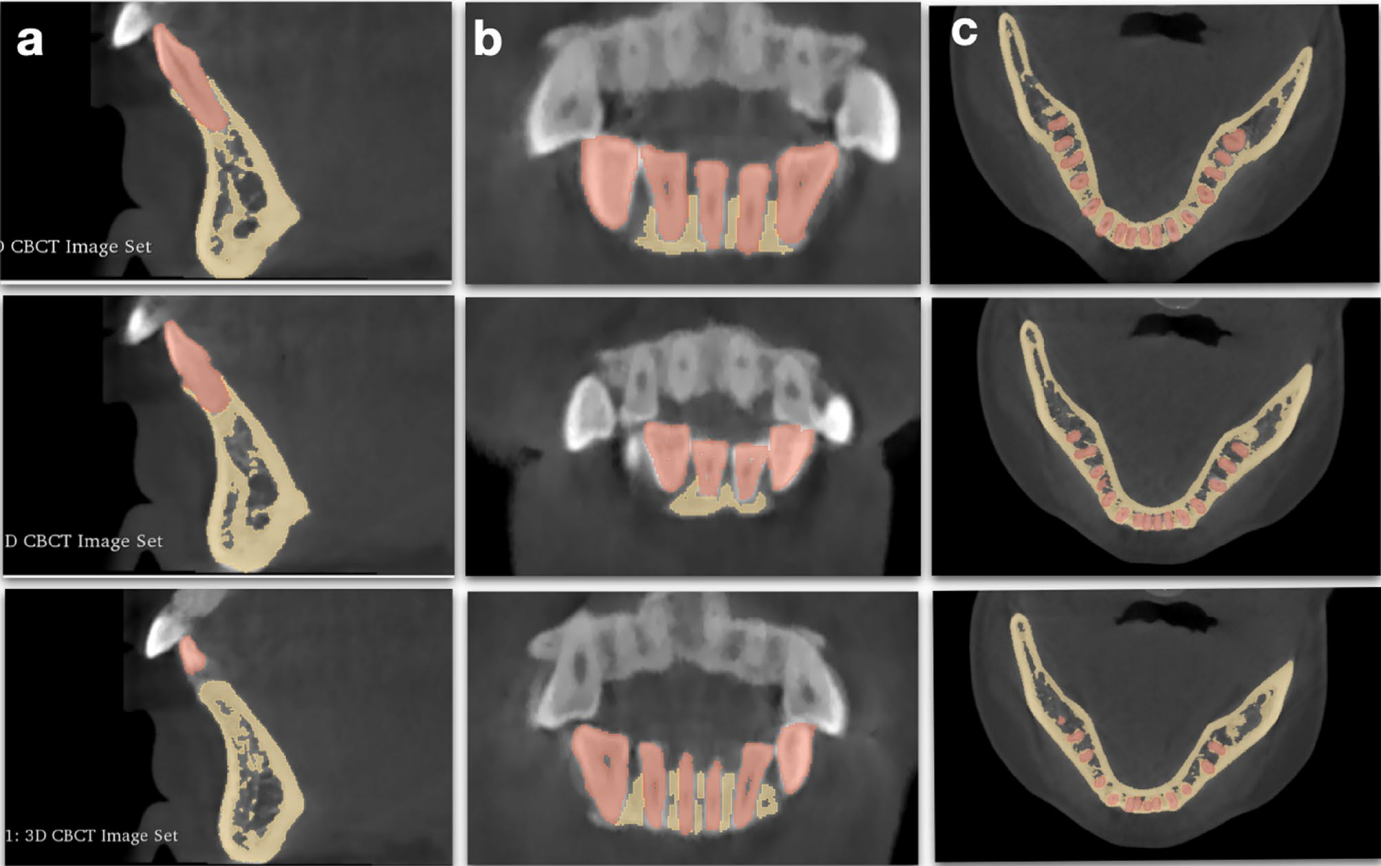
Regions of interest (ROIs) highlighted on slices of the CBCT dataset in 3D slicer software (Yellow: mandible; Orange: teeth). (**a**) Sagittal view. (**b**) Coronal view. (**c**) Axial view.

All CBCT scans at T1 and T2 were taken by the same CBCT machine, NewTom VGI (Quantitative Radiology, Verona, Italy) with the following settings: field of view (FOW), 200 × 200 mm^2^, 90 kV; 6.0 mA; scan time, 15 s; and voxel size, 0.3 mm. The volume data were exported in Digital Imaging and Communications in Medicine (DICOM) format. The pre- and post-treatment mandibles were segmented, including Pog and the mental foramen, and converted to STL files using 3D Slicer software (version 4.9.0; www.slicer.org), a free, open-source software application for medical image computing^17^. Regions of interest (ROIs) were constructed to represent the mandible and teeth that were distinct from one another. A variety of semi-automatic and manual segmentation tools were used in the process (Fig. 1). First, the DICOM images were filtered using Gradient Anisotropic Diffusion for denoising. Next, the “Level Tracing tool” (which is used to trace a region where all pixels have the same background value as the selected pixel) was applied to the mandible and teeth on every third slice, starting at the right side and working all the way to the left side for the mandible, and starting at the mesial side and working our way to the distal side of each tooth. Afterward, “Fill Between Slices” was used to generate the missing ROIs, with morphological interpolation used to construct the remaining ROIs. Individual ROIs were further refined using hand segmentation procedures, allowing even more precise results (Paintbrush, Erase, Scissors). Finally, The ROIs were used to produce three-dimensional polygon models, which were then exported as STL files.

#### Preparation of ClinCheck models

The initial and final STL files of the first series of aligner treatment were exported from ClinCheck software directly with a dental superimposition feature. The initial STL file from the refinement ClinCheck was also exported separately as T2 digital model. Rhinoceros software (version 6.0; www.rhino3d.com; Robert McNeel & Associates, Seattle, WA, USA) was used to segment only the occlusal one-third of the clinical crowns on the ClinCheck models to improve the precision of superimposition by using a smaller and reliable part of the tooth structure.

#### Model registrations and superimposition of T1 and T2 CBCT

**Figure 2:**
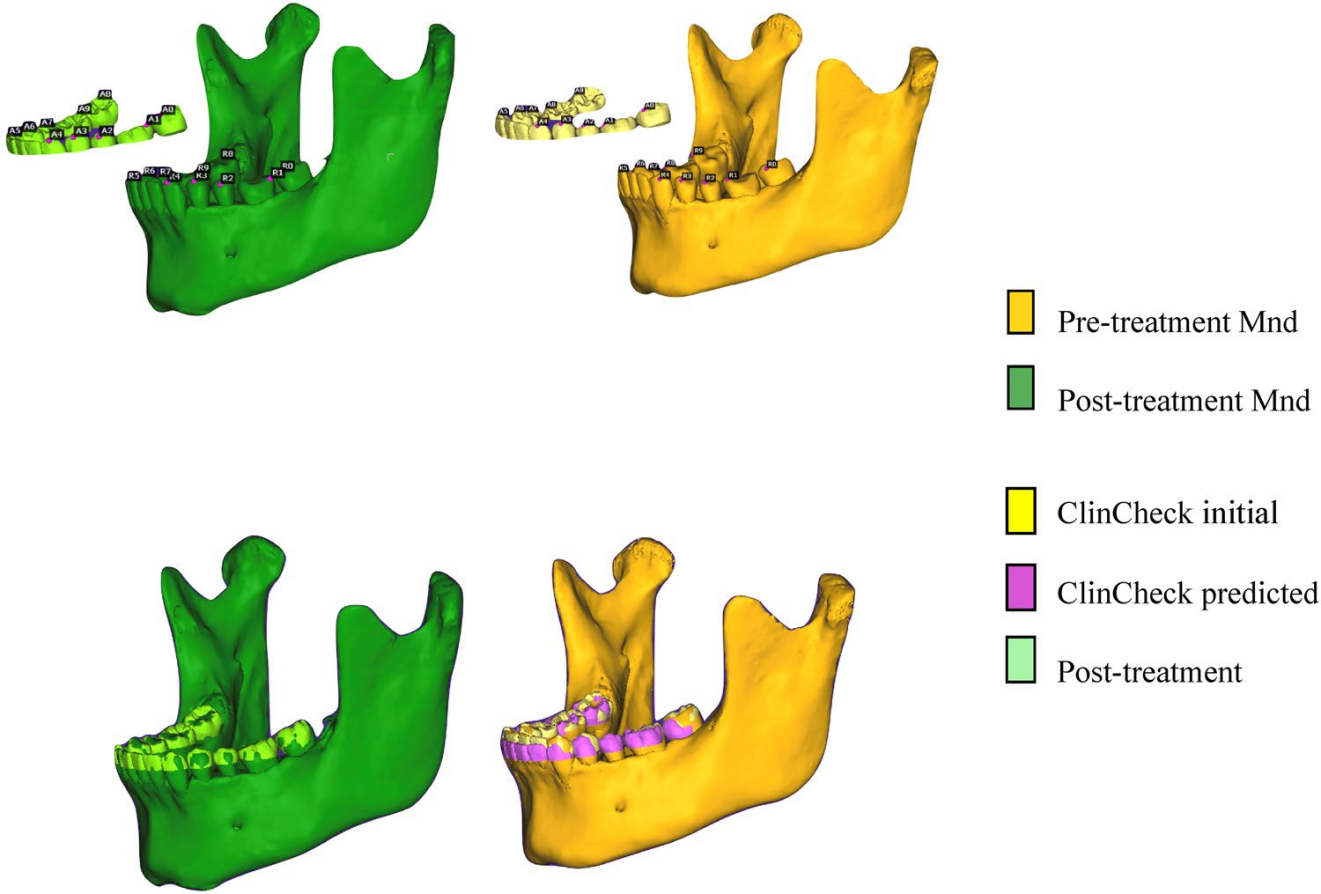
T1 (ClinCheck initial of the first series) and T2 (ClinCheck initial of the refinement series) models aligned with their corresponding mandibles using CloudCompare along with the ClinCheck predicted model.

The STL files of the segmented mandible with dentition from T1 and T2 CBCT, ClinCheck initial and final model, and the T2 actual model were imported into CloudCompare software (version 2.11, GPL software, retrieved from http://www.cloudcompare.org) and aligned accordingly. The ClinCheck initial model was registered on the dentition part of T1 CBCT model together with ClinCheck predicted model, and T2 actual model was integrated with T2 CBCT model based on dentition superimposition (Fig. 2).

**Figure 3:**
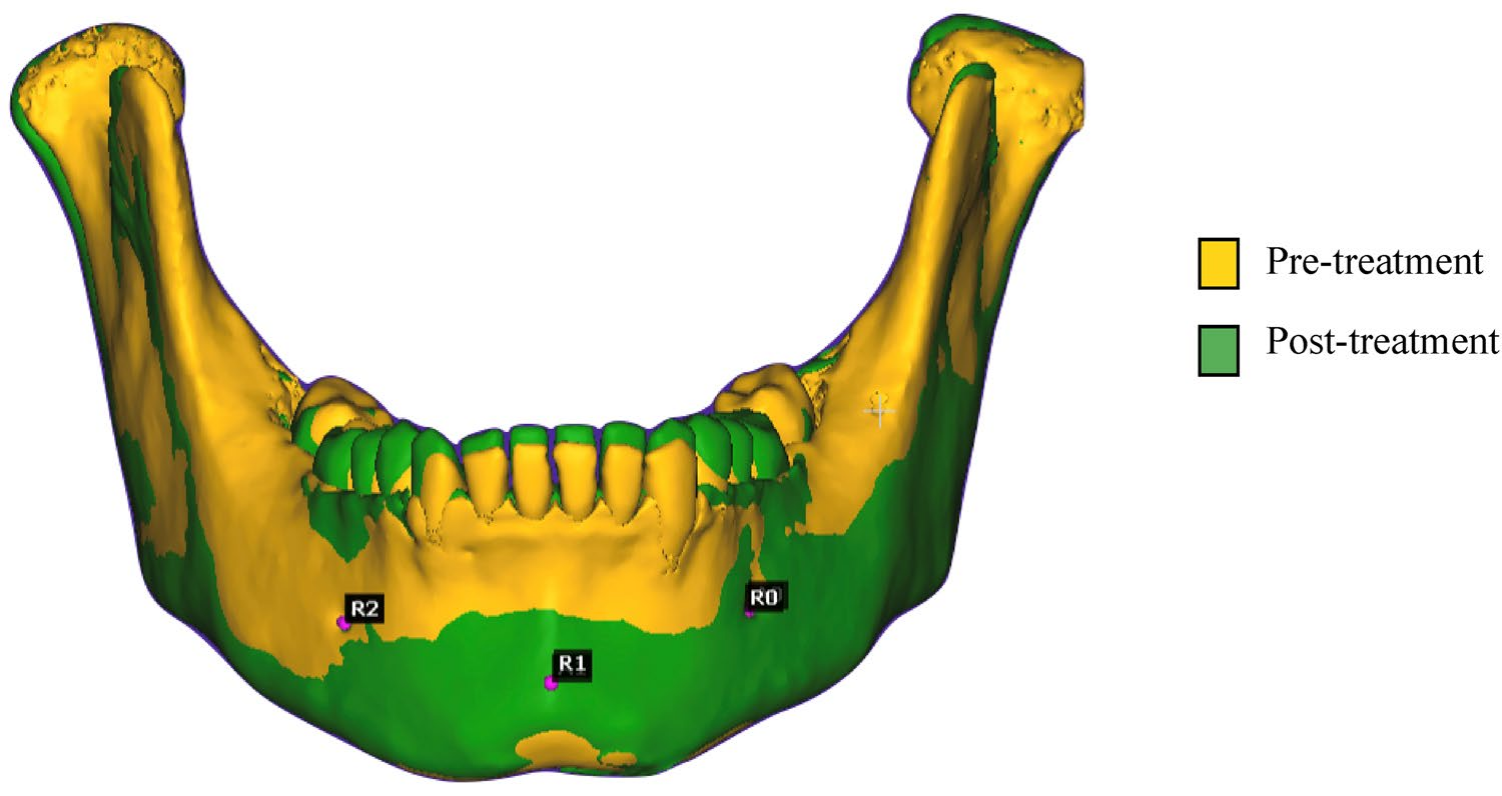
Pre- and post-treatment mandibular superimposition with rough alignment in CloudCompare.

**Figure 4:**
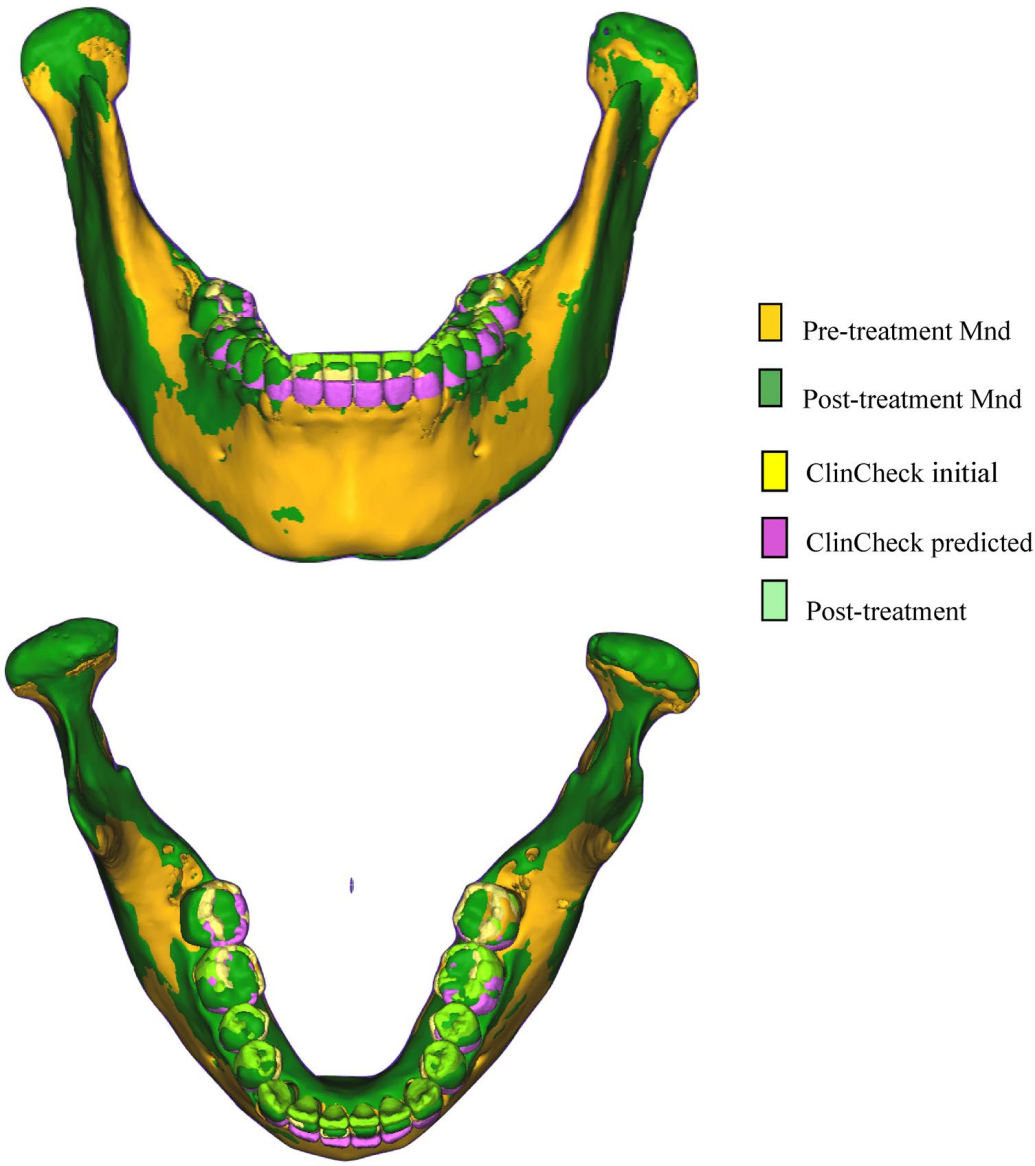
Pre- and post-treatment mandibular superimposition after fine alignment in CloudCompare.

Rough alignment of T1 and T2 CBCT models (Fig. 3) was registered on the relatively stable landmarks, Pog (the most anterior point in mandibular chin area in the sagittal plan) and bilateral mental foramen on each model, followed by fine alignment (Fig. 4) with iterative closest point (ICP) tool^18^. CloudCompare outputs the resulting transformation matrix in a console and displays the registration information window that describes the Root Mean Square (RMS) RMS. The acceptable deviation from the perfect fit was RMS ≤ 0.05.

**Figure 5:**
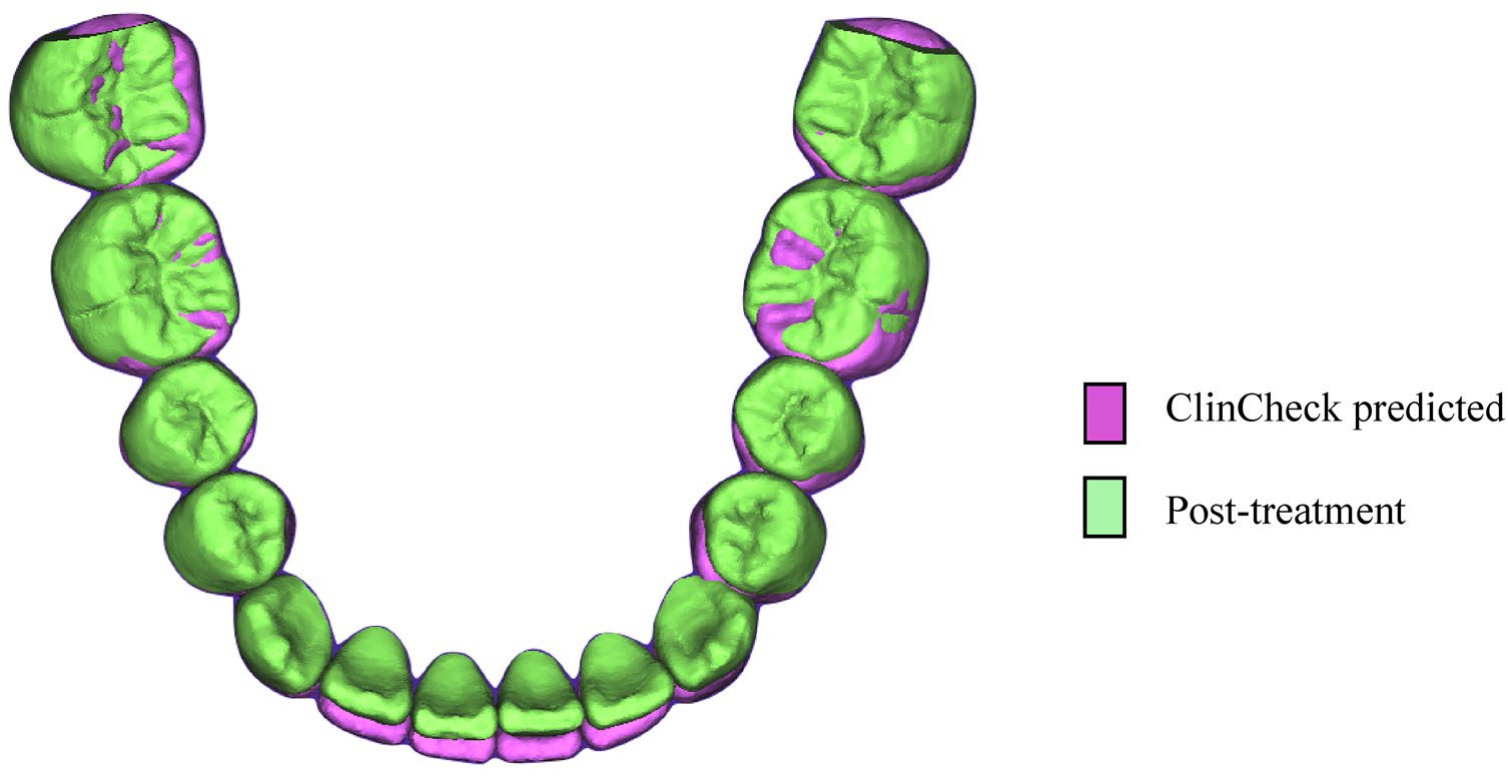
Superimposition of ClinCheck predicted and post-treatment actual model in CloudCompare.

With the request to hide all models except the ClinCheck predicted and T2 actual model, the final superimposition of the actual and predicted mandibular models were obtained (Fig. 5).

### Measurement of the prediction difference

**Figure 6:**
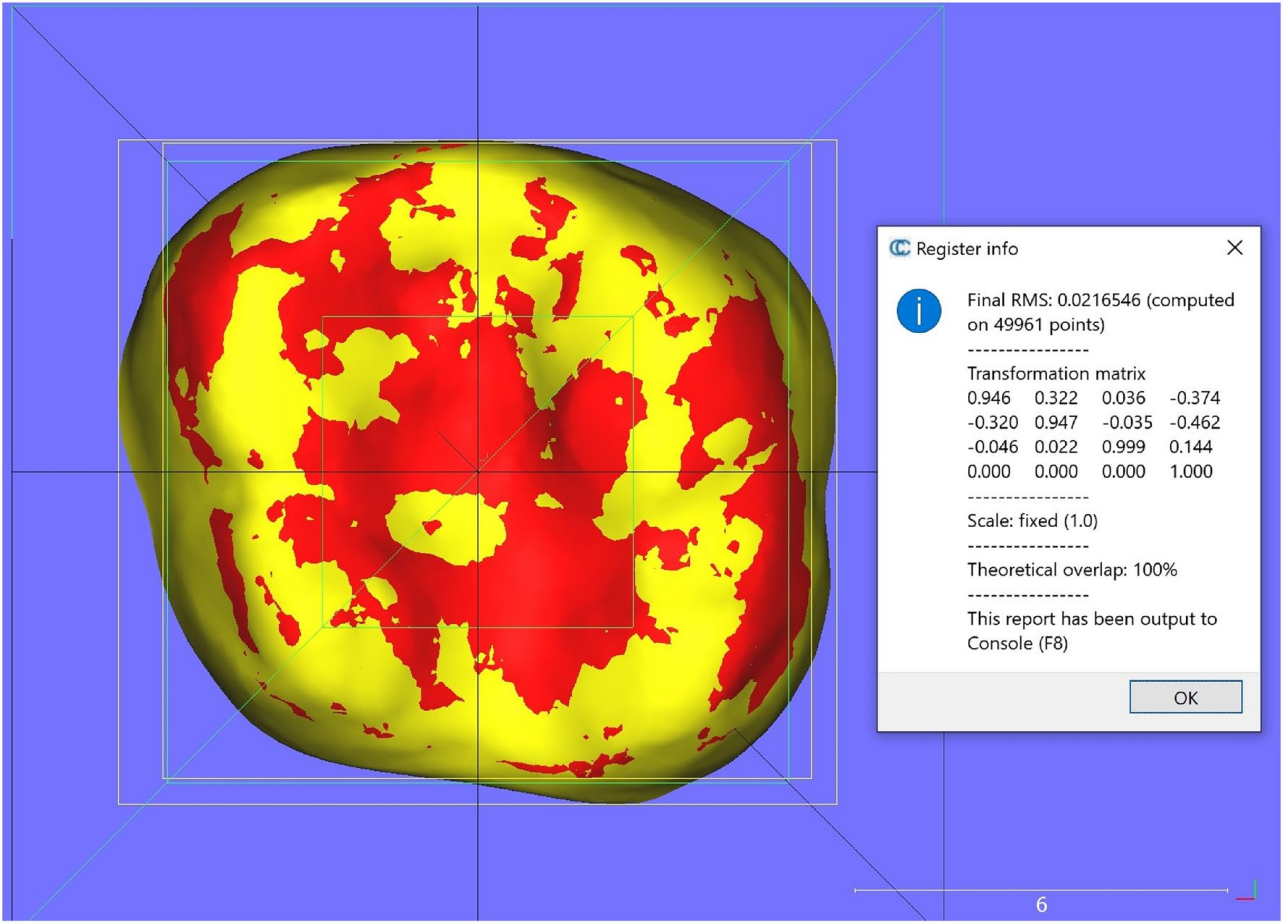
Superimposed ClinCheck predicted tooth over the post-treatment tooth with registration information window in CloudCompare.

**Figure 7:**
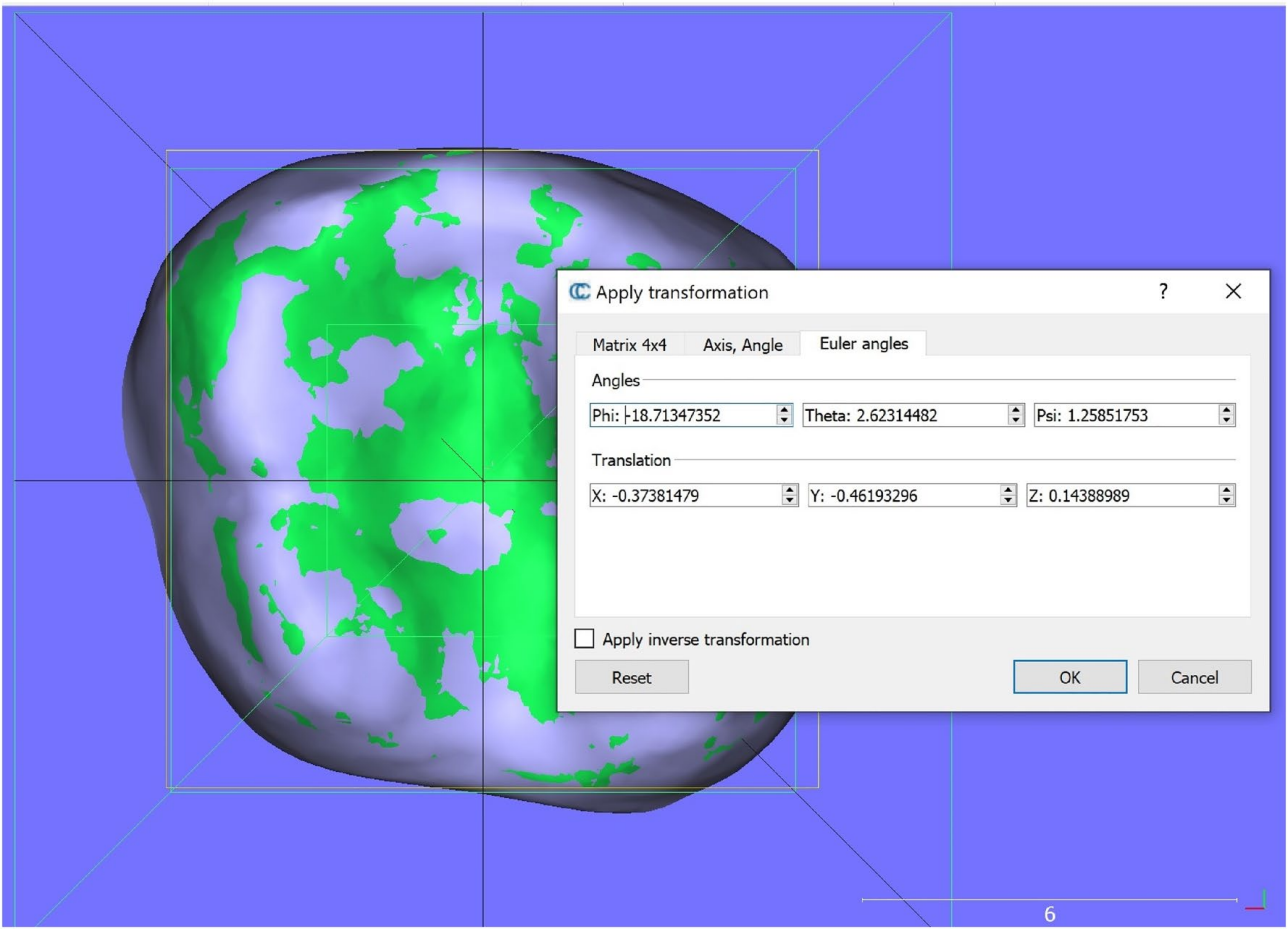
Values for the 3D measures derived from the transformation matrix with CloudCompare.

To assess the 3D tooth positional difference of each mandibular tooth between the superimposed achieved model and ClinCheck predicted model, the single tooth registration was also performed in CloudCompare, each fit providing the transformation needed to move that tooth from its achieved to its predicted position. Each movement was expressed relative to a world coordinate system. We used a cuboidal template within which the surface of the achieved tooth crown was oriented so its occlusal and facial surfaces aligned with the world axes, and its bounding-box center coincided with the world origin. An example of the individual tooth registration is shown in Fig. 6, which includes the transformation matrix, the RMS indicating the goodness-of-fit, the three Euler angles and the three axial translations derived from the matrix (Fig. 7). Differences larger than 0.5 mm for linear and 2 degrees for angular measurements would be considered clinically relevant according to the Objective Grading System of the American Board of Orthodontics (ABO-OGS) for a case evaluation.

**Figure 8:**
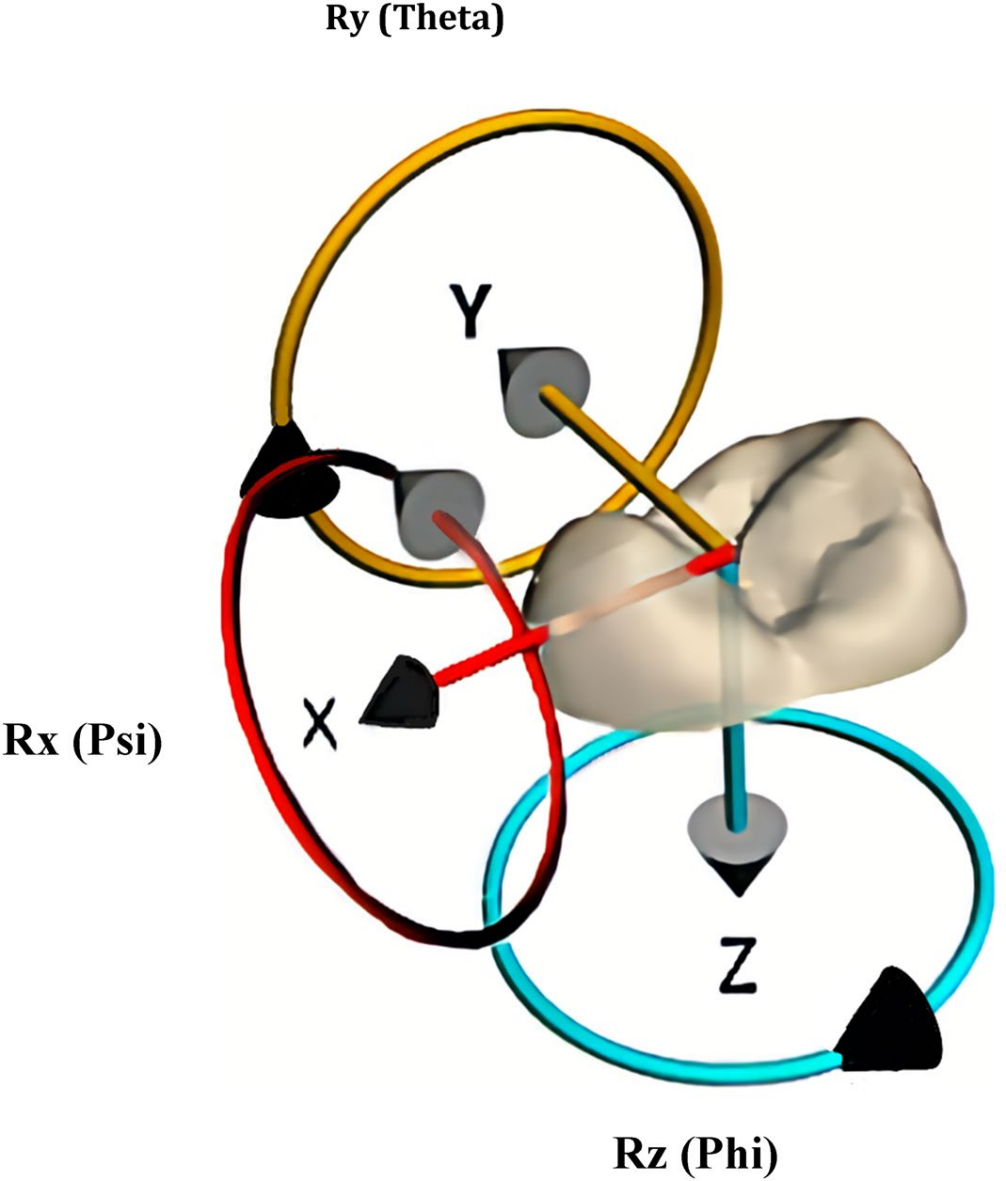
The axes and Euler angles used in this study, including the buccal-lingual axis X, the mesial-distal axis Y, and the occlusal-gingival axis Z. Rx (Psi), or tip represents rotation around X axis; Ry (Theta) or torque represents rotation around Y axis and Rz (Phi), represents rotation around the vertical Z axis. The axes are referenced to the premolar’s anatomical surfaces.

In Fig. 8, it shows the world coordinate system and Euler angles used in this study: translation along the X‑axis represents buccolingual movement, along the Y‑axis mesiodistal movement, and along the Z‑axis occlusogingival movement; while rotation around the X-axis (Psi) represents mesiodistal tipping, around the Y-axis (Theta), buccolingual torque, and around the Z-axis (Phi) mesiodistal rotation. All the 6 measures of tooth orientation account for directionality. For example, for a single tooth orientation measure, the predicted tooth orientation acts as the origin (and is hence 0 for that measure) while an observed or achieved tooth orientation can be positive or negative along that measure. The recorded prediction difference is thus the change required by the observed tooth to reach the origin. So if the observed tooth orientation is positive with respect to the origin, then the corresponding prediction difference is negative, and vice versa.

The differences between the predicted and achieved tooth position of these seventy teeth from 5 patients were measured and grouped into four groups according to different tooth types: incisor, canine, premolar and molar.

### Statistical analysis

All measurements of the 70 teeth were performed by one examiner (A.A.). We randomly selected six teeth and reassessed by the same and a second examiner. Intra- and inter-examiner reliability were tested using intraclass correlation coefficients (ICC) and Bland–Altman analyses.

One-sample *T* test was used to test whether or not the sample mean of a sample is significantly different from a pre-specified mean (here it is zero). To adjust for multiple tooth comparisons, we applied a Bonferroni correction to the p-values and the P value was set at 0.05. The analyses were performed using the R statistical package (v 3.2.3, RStudio Inc., Boston, Mass) through RStudio (version 1.4.1103).

### Ethical approval

All procedures performed in studies involving human participants were in accordance with the ethical standards of the institutional and national research committee and with the 1964 Helsinki declaration and its later amendments or comparable ethical standards. The study was reviewed, informed consent was waived and the research protocol was approved by the Institutional Research Board at University of British Columbia, Vancouver, Canada (No. H21-02474).

### Informed consent

Since this is a retrospective study, inform consent was waivered. All data collected were de-identified.

## Results

Table 1 displays the corrected p-values for the tests, and one can see that many of the tests are not statistically significant. The only evidences of non-zero prediction difference means are for “Premolar” Phi, “Incisor” Psi and “Molar” Y, at the 5% significance level.

**Table 1:** Prediction difference means *P* value with one sample *t* tests.

Tooth type	Phi	Theta	Psi	X	Y	Z
Incisor (n = 20)	1.00000	1	0.04030*	1	1.00000	1
Canine (n = 10)	1.00000	1	1.00000	1	1.00000	1
Premolar (n = 20)	0.01312*	1	0.93025	1	0.56104	1
Molar (n = 20)	1.00000	1	1.00000	1	0.04471*	1

Adjusted for multiple testing.

*P < 0.05.

**Figure 9:**
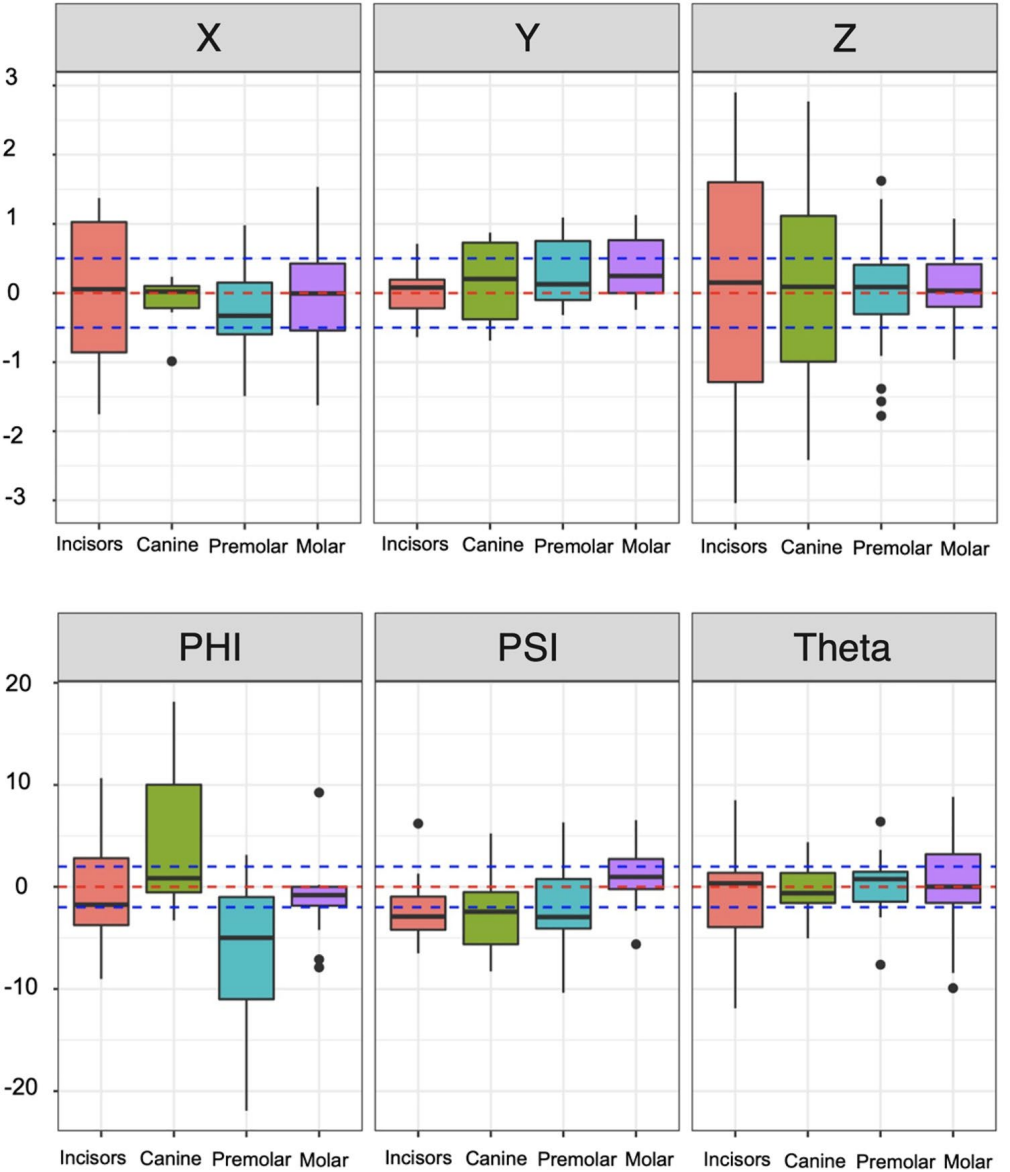
Tooth measure prediction differences across tooth type.

Tables 2 and 3 provide 95% confidence intervals of the mean prediction difference for each tooth measure, tooth type combination. We can also see that the specific 95% confidence intervals which do not contain 0 correspond to the tests that were statistically significant. The “Premolar” Phi and “Incisor” Psi mean prediction differences appear to be biased in the negative direction, while the “Molar” Y mean prediction difference is biased in the positive direction. These results somewhat corroborate what is observed in the prediction difference boxplots (Fig. 9).

**Table 2:** 95% confidence intervals of angular tooth measures (with Bonferroni Correction).

Tooth type	Phi		Theta		Psi	
	Lower bound	Upper bound	Lower bound	Upper bound	Lower bound	Upper bound
Incisor (n = 20)	− 4.397	3.928	− 4.129	3.281	− 4.773	− 0.063
Canine (n = 10)	− 5.299	14.948	− 4.533	3.961	− 8.368	3.043
Premolar (n = 20)	− 12.287	− 0.935	− 2.225	2.453	− 5.139	1.192
Molar (n = 20)	− 3.939	1.484	− 3.423	4.095	− 1.194	2.998

**Table 3:** 95% confidence intervals of linear tooth measures (with Bonferroni Correction).

Tooth type	X		Y		Z	
	Lower bound	Upper bound	Lower bound	Upper bound	Lower bound	Upper bound
Incisor (n = 20)	− 0.859	0.778	− 0.251	0.304	− 1.449	1.509
Canine (n = 10)	− 0.583	0.384	− 0.674	0.963	− 2.131	2.253
Premolar (n = 20)	− 0.716	0.359	− 0.119	0.656	− 0.721	0.716
Molar (n = 20)	− 0.722	0.489	0.005	0.682	− 0.279	0.534

**Figure 10:**
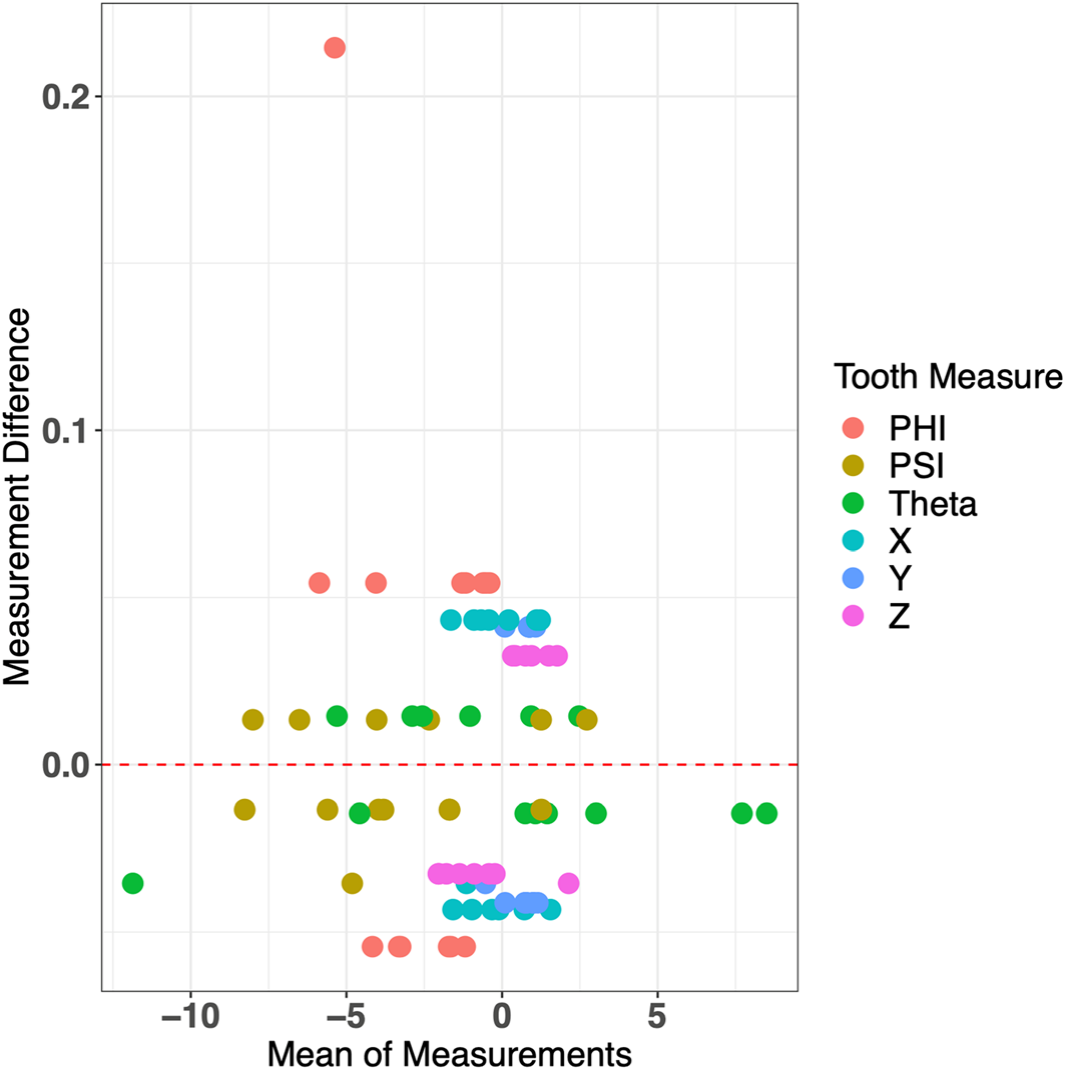
Bland–Altman plot for inter-examiner agreement.

The ICC value for inter-examiner agreement is very high, with a value of 0.996, which nearly reaches 1. Examining the Bland–Altman plot (Fig. 10), we see that the majority of points cluster near 0 for both the mean of measurements and the measurement differences. We do see one observation that has a very large positive measurement difference, corresponding to one of the Phi measurements. Notably, the measurement differences for the Phi tooth measure lie mostly on the positive side of the 0 line (outside of the outlier previously addressed). This suggests that the second examiner’s set of Phi measurements exceeds the original set of Phi measurements with some consistency. In general, though, the inter-examiner agreement is very high.

**Figure 11:**
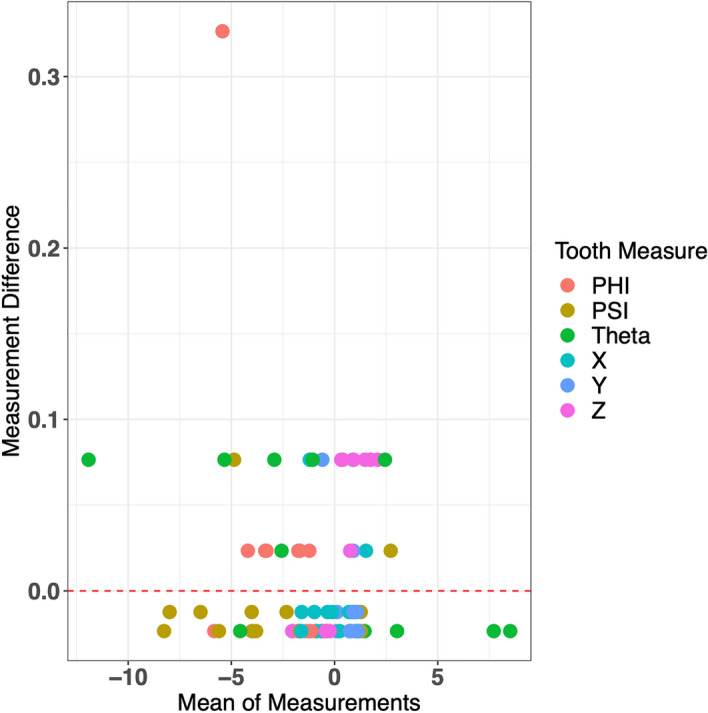
Bland–Altman plot for intra-examiner agreement.

The ICC value for the intra-examiner agreement is also very high, with a value of 0.999. The Bland–Altman plot (Fig. 11) does not show any clear patterns between points. We once again observed that there is a large positive outlier for a single Phi measurement difference, and measurement differences, in general, are very low, across means of measurements. This suggests that overall, there is a very high agreement between the original set of measurements and the second set of measurements made by the original examiner.

## Discussion

Previously, several studies used different assessment tools to evaluate the effectiveness of clear aligner therapy. For example, the Toot0068Measure program (Align Technology)^19–22^, the Orthodontics Model Grading System^23,24^, CBCT^21,25^, or Simulator Software^26^. These assessment tools may provide a general assessment when lacking a stable reference structure for superimposition. In the present study, we used multiple software for different purposes.

3DSlicer was used to segment CBCT images. CloudCompare was used to measure the linear and angular difference between objects with respect to the six components of the transformation matrix (X, Y, Z, Phi, Theta, Psi). In the present study, it is essential to discuss the CouldCompare registration process to interpret the findings appropriately. To measure the difference between the ClinCheck predicated and actual tooth movement, we used the transformation matrix approach. This approach provides a standardized metric to measure the difference between the ClinCheck-predicted tooth and the actual post-treatment tooth.

Cartesian coordinate systems relative to an object in space can be extrinsic or intrinsic. A World Coordinate System is extrinsic, and a Local one is intrinsic. The numerical descriptors needed to the change in pose according to a coordinate system include displacements along, and rotations around the three defined axes that pass through a common origin, so they could be described within a World or Local frame. CloudCompare always uses the platform’s World Coordinates for all calculations; however, calculations based on Local coordinates depend on user-specification of that object’s origin and axes and are typically based on its physical features. If the moving object is not located at the World’s origin, any rotations around it alter the object’s position in World space in addition to any translational movement. This unwanted effect can be avoided if the object is initially centred on the World origin and aligned to World axes before it is moved, and effectively creates a Local coordinate system that is coincident with the World one. In our registration sequence, both predicted and achieved teeth were posed initially and the tooth to be moved (predicted tooth) was positioned at CloudCompare’s origin, and its known anatomical features coincided with CloudCompare’s axes (Fig. 8). The software then used the Tait-Bryan convention for calculating rotations and displacements, so its transformation matrix expressed all variables with respect to both World and Local coordinates.

Invisalign system provides transformation measurements that describe the difference between the ClinCheck pre-treatment and predicted teeth; however, we are not aware of the convention system that Invisalign uses for their measurements. Therefore, comparing their measurement data with our findings wouldn’t be a valid option for our analysis. Moreover, the coordinate system could be utilized differently for measurements, such as using a transformation matrix based on a tooth-based Cartesian system or measuring tooth-orientations expressed as lines, axes or vectors (polar coordinate system).

Overall, the results of this study showed that the mean prediction differences of X (buccal-lingual transitional tooth movement), Z (vertical tooth movement), and Theta (buccolingual tooth movement (torque)) are not statistically different across all teeth types (Table 1).

In the present study, the premolars showed the only statistically significant prediction difference with Phi (rotation tooth movement). Rossini and colleagues published a systematic review in 2015 to evaluate the efficacy of clear aligner treatment in controlling orthodontic tooth movement. They reviewed relevant studies starting from 2000 to mid-2014. They found that rotation tooth movement is challenging, with an accuracy of 36%^27^. In addition, Simon et al. conducted a study to assess the efficacy of an aligner technique regarding incisor torque, premolar derotation, and molar distalization. They found the lowest accurate tooth is premolar derotation with 40%^28^. One might think this partly due to the old aligner materials involved. However, in our study, we included only cases that were treated with the newer Invisalign aligner material (SmartTrack) and also found that derotation of the 1st premolar is less accurate with Invisalign system.

In terms of Y (mesiodistal transition movement), in our study, we found a statically significant difference in mesiodistal transitional movements between the molars of the ClinCheck predicted model and the post-treatment model superimposition (in the positive direction). This indicates that the positions of the achieved post-treatment teeth were more distally in relation to the ClinCheck predicted teeth. These findings agree with the efficacy of Invisalign for distalizing molars^28^. However, our findings didn’t show an accuracy between the predicted and achieved distalization. We also found that there is a statically significant difference in the Psi (mesiodistal tipping movement) between the incisors of the ClinCheck predicted model and the post-treatment model superimposition. This is different from Lombardo et al.^29^ work indicating that mesiodistal tipping is quite accurate. This difference could be explained either by the different clear aligner system (F22 aligners system, Sweden & Martina, Due Carrara, Italy) or measurement methodology, with sample size discrepancy as another potential factor.

Some strengths could be associated with this study. First, this study used a novel methodology utilizing the mandibular body to superimpose the ClinCheck initial model along with its final predicted model over the post-treatment models. Second, utilizing accurate superimposition software such as CloudCompare would minimize the risk of measurement bias. This study presents some limitations, such as the small sample size, which is expected due to the difficulty to obtain pre-and post-treatment (at refinement) CBCT images for orthodontic patients. This is mainly due to the need for justification for taking images with high radiation doses at pre- and post-treatment (refinement) time points. Recently, Align Tech has added the CBCT feature to their ClinCheck. Hopefully, with the future introduction of low-dose radiation CBCT, it might be feasible to assess the tooth movement, including the roots and their relation to the basal bone.

Another limitation is the retrospective nature of the study, which makes the selection bias a possibility. In addition, patient compliance was difficult to control in our sample collections. Additional limitation of this study, which applies to studies with a similar design, is assessing the tooth movements of several teeth on the same patients. This would question if the tooth movements are induced mainly by the effect of aligners, or also by the effect of the adjacent teeth movements. In theory, to avoid this limitation, one tooth and one tooth movement should be measured in each participant, which will require an ample sample size or fewer tooth movements to measure. Alternatively, using statistics that account for matching and clustering effects, such as multivariate models, would be an optimal solution. However, this would require an adequate sample size as well. Finally, the resultant tooth movements could be influenced by the experiences of the clinicians with the treatment plan using Invisalign. Furthermore, using multiple software makes the process complicated and prone to errors. Each model segmentation and measurement would take up to a few hours to complete. Finally, as the best fit technique is used, the differences between the predicted and achieved tooth position may appear small, but they do not represent the prediction position of the teeth within the bone and in the face.

## Conclusion

In this study we aimed to demonstrate a robust novel methodology which can be employed to quantify 3D tooth positional difference either with orthodontic treatment or with growth by using CBCT and dental superimposition based on stable anatomic landmarks. However, the findings in terms of the prediction of Invisalign treatment in the mandibular teeth in non-extraction cases are potentially weak, which merits further and more rigorous investigations not only due to the limited sample size but also because that ClinCheck is more of a tool to design clinicians’ biomechanics rather than merely a tool for visualization of the predicted treatment outcomes when planning clear aligner therapy.

## Data Availability

The datasets used and/or analysed during the current study available from the corresponding author on reasonable request.
